# Successful immune checkpoint inhibitor‐based rechallenge in a patient with advanced esophageal squamous cell cancer: A case report

**DOI:** 10.1111/1759-7714.14279

**Published:** 2022-01-11

**Authors:** Yanhong Yao, Zhentao Liu, Qian Li, Baoshan Cao, Mopei Wang

**Affiliations:** ^1^ Department of Medical Oncology and Radiation Sickness Peking University Third Hospital Beijing China

**Keywords:** esophageal squamous cell cancer (ESCC), immune checkpoint inhibitors (ICIs), rechallenge, tracheoesophageal fistula (TEF)

## Abstract

Immune checkpoint inhibitors (ICIs) have been shown to improve survival in patients with advanced or metastatic esophageal cancer. However, ICI‐based rechallenges after recovery from fatal adverse events (AEs) are equivocal, especially in patients who have already undergone treatment‐related AEs. In this study, we report the case of a patient with advanced esophageal squamous cell cancer (ESCC) who developed a treatment‐related tracheoesophageal fistula (TEF) after two cycles of ICI administration, provided in combination with traditional chemotherapeutics. After spontaneous healing of the TEF, the patient was again treated with ICIs and achieved a durable clinical response without any signs of fistula recurrence. Successful ICI‐based rechallenges after fistula healing have rarely been reported. Therefore, ICI‐based rechallenge in patients with esophageal cancer having an Eastern Cooperative Oncology Group (ECOG) performance status (PS) 0–1 after serious AEs may serve as a clinically viable treatment strategy that should be administered under close monitoring.

## Graphical abstract

Immune checkpoint inhibitors (ICIs) have been shown to improve survival in patients diagnosed with advanced or metastatic esophageal cancer. However, ICI‐based rechallenges after recovery from fatal adverse events (AEs) are equivocal, especially in patients who have already undergone treatment‐related AEs. In this study, we report the case of a patient with advanced esophageal squamous cell cancer (ESCC) who developed a treatment‐related tracheoesophageal fistula (TEF) after two cycles of ICI administration, provided in combination with traditional chemotherapeutics. After spontaneous healing of the TEF, the patient was again treated with an ICI. The patient acquired a durable clinical response without any signs of fistula recurrence. Successful ICI‐based rechallenges after fistula healing are rarely reported. Therefore, ICI‐based rechallenge in patients with esophageal cancer having an Eastern Cooperative Oncology Group (ECOG) performance status (PS) 0–1 after serious AEs may serve as a clinically viable treatment strategy that should be administered under close monitoring.

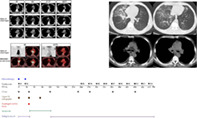

## INTRODUCTION

The global cancer statistics of 2020 indicated that esophageal cancer is the seventh most common form of malignancy and represents the sixth leading cause of cancer‐related deaths worldwide.^1^ The highest regional incidence of esophageal cancer has been reported in Eastern Asia, especially in China, where esophageal squamous cell carcinoma (ESCC) accounts for 95% of the total cancer incidences.^2^ Chemotherapeutic agents, such as taxane, fluorouracil, and cisplatin, are commonly used as first‐line treatment for patients with advanced or metastatic ESCC. To date, several studies have reported enhanced efficacies and manageable safety profiles of established chemotherapeutic agents used in combination with immune checkpoint inhibitors (ICIs), such as anti‐programmed cell death protein 1 (PD‐1) antibodies as first‐line treatment for ESCC patients.^3^, ^4^

However, the use of ICIs is accompanied with toxicity. Although most adverse events (AEs) can be categorized generally as grade 1 or 2, a small number of them are serious and life‐threatening.^5^ A few serious adverse events (SAEs) in esophageal cancer patients such as an esophageal leak, fistula formation, and esophageal perforation may happen during tumor remission.^6^ The role of ICI‐based rechallenge remains undefined in cancer patients for whom ICIs have been discontinued due to toxicity‐associated side‐effects.

In this study, we report the case of a 56‐year‐old Chinese male with advanced ESCC who received tislelizumab, a PD‐L1 inhibitor, in combination with traditional chemotherapeutic agents as first‐line treatment. After two cycles of therapy, he suffered from a tracheoesophageal fistula (TEF). A tislelizumab‐based rechallenge therapy after spontaneous healing of TEF led to a better clinical outcome in the patient.

## CASE REPORT

The patient was a 56‐year‐old male who initially presented with a history of dysphagia since June 2020, with no other symptoms, such as chest pain, weight loss, and fatigue. He had a history of stones in his right kidney with dilatation in the right renal pelvic area, with creatinine levels maintained at the upper limit of normal. The Eastern Cooperative Oncology Group (ECOG) performance status (PS) of the patient was 1. Gastroscopic examination showed an irregular‐shaped bulging tumor, located 24–34 cm away from the incisor teeth, invading nearly the entire circumference of the esophagus, and obstructing the passage of the gastroscope. Pathological analyses were positive for ESCC, while the status of expression of the programmed death ligand‐1 (PD‐L1) protein in the tumor tissue was unknown. Upper gastrointestinal radiography showed severe stenosis in the upper‐middle esophagus with a range of about 10 cm. The fluorodeoxyglucose (FDG)‐positron emission tomography‐computed tomography (PET‐CT) scan revealed that a upper‐middle esophageal tumor was invading the carina and right hilar lymph node, and tracheoesophageal groove lymph node‐based metastasis was also evident. This patient was staged as cT4bN + M0 (IVA), based on the guidelines in the eighth edition of the American Joint Committee on Cancer (AJCC) staging manual.

**FIGURE 1:**
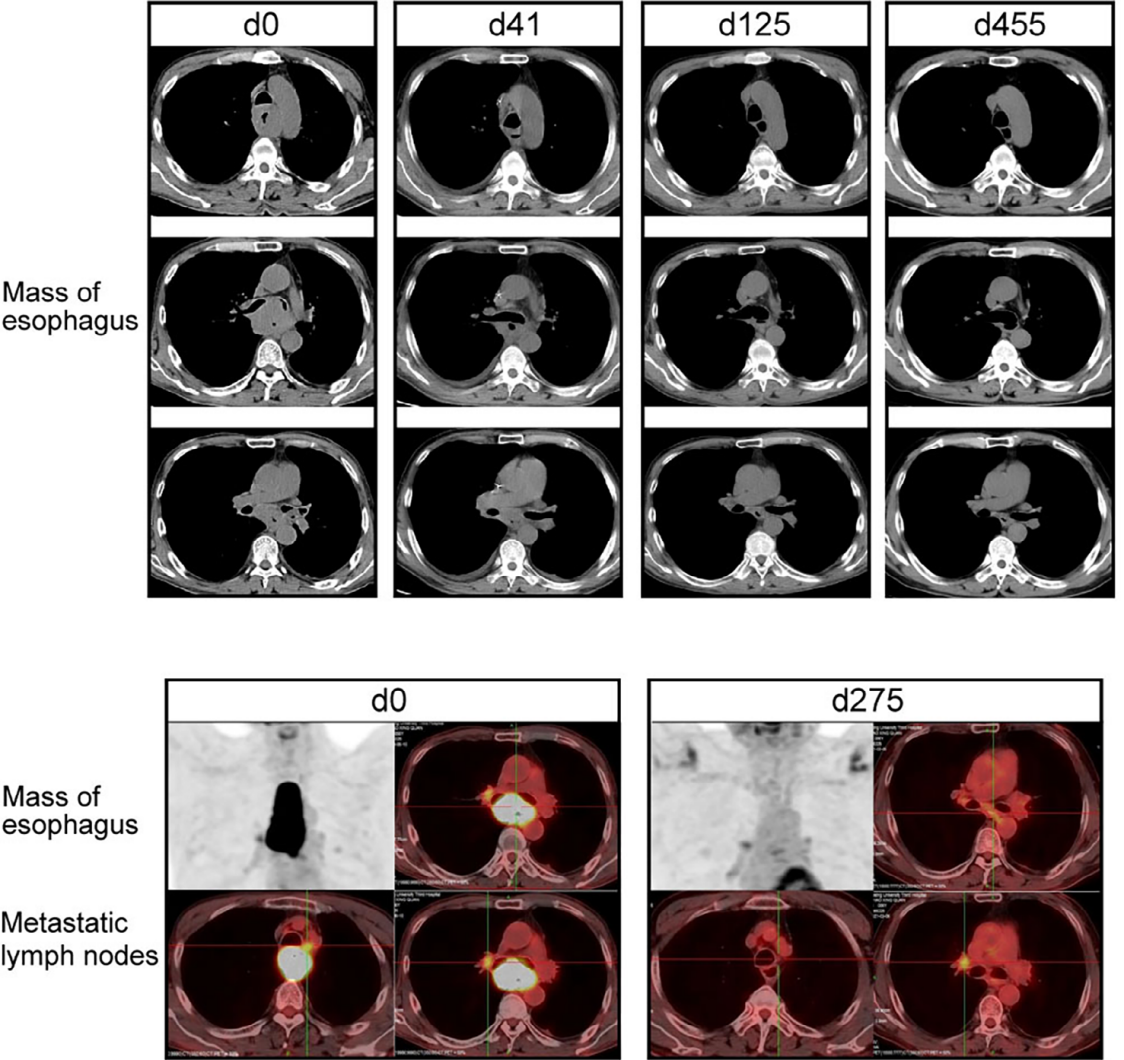
Monitoring tumor response to treatment. Representative computed tomography (CT) and fluorodeoxyglucose (FDG)‐positron emission tomography‐computed tomography (PET‐CT) images of tumor before and after therapy

**FIGURE 2:**
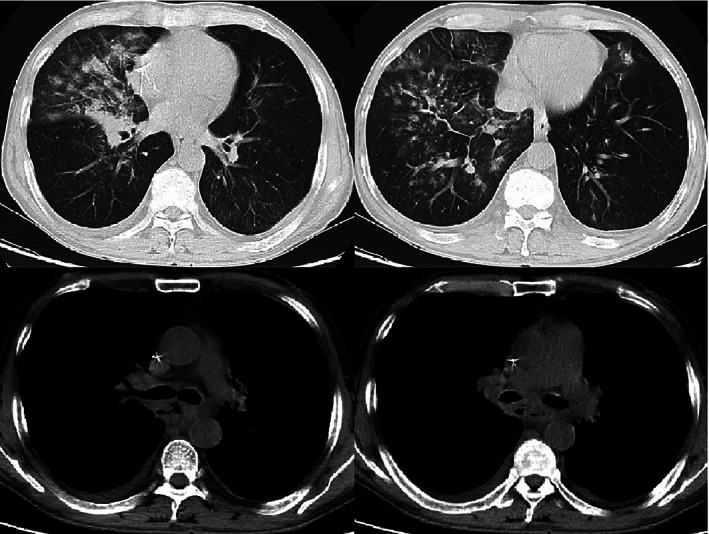
Representative CT images of pneumonia and tracheoesophageal fistula

The patient received two treatment cycles of tislelizumab 200 mg and chemotherapy with albumin‐bound paclitaxel 125 mg m^−2^ 200 mg on days 1 and 8, and nedaplatin 80 mg m^−2^ 120 mg on day 1 every three weeks since June 18, 2020. He experienced grade one nausea and grade three neutrophil count decrease, which improved rapidly after the introduction of the new therapeutic intervention. In response to the first cycle of therapy, the patient no longer presented with dysphagia. However, there was a sudden onset of fever and an elevated level of procalcitonin was observed, without any other additional symptoms, as documented on July 27, 2020, the 41st day of treatment. Emergency CT scan showed significant esophageal tumor regression (Figure 1). However, a small zone of free air near the middle esophagus and a localized patchy shadow in the right lung lobe were observed (Figure 2), and TEF accompanied with aspiration pneumonia was highly suspected. Upper gastrointestinal radiography revealed the presence of a fistula (length, 5 mm) between the mid‐esophageal region and the trachea (Supplementary Material S1). A comprehensive treatment regimen of proton pump inhibitor and antibiotics was started and nasointestinal tube for enteral nutrition (EN) support was introduced. The body temperature and procalcitonin normalized over a period of 2 weeks. Upper gastrointestinal radiography (Supplementary Material S2) confirmed the disappearance of the fistula on September 9, 2020, 6 weeks after its appearance. A CT scan on October 21, 2020 revealed further tumor regression even in the absence of any anticancer treatment for over 3 months since the fistula was diagnosed, and the fistula had healed completely (Figure 1). The nasointestinal tube was removed so the patient could resume oral ingestion of food.

**FIGURE 3:**
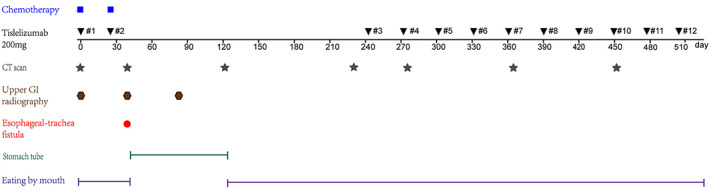
Summary of the clinical treatment of the patient

Taking the positive outcome of the previous drug treatment and stable health condition being maintained by the patient into consideration (Figure 3), we decided to administer a rechallenge therapeutic regimen of 200 mg tislelizumab every 4 weeks under close monitoring on February 20, 2021, after more than 6 months of the occurrence of the fistula, following the clinical decision of the multidisciplinary team (MDT). PET/CT scan did not reveal any signs of malignancy in the esophagus as of March 20, 2021 (Figure 1). To date, routine examinations conducted every 3 months have shown no signs of cancer progression and the patient has an ECOG PS of 0.

## DISCUSSION

Combinatorial therapy involving anti‐PD‐1 antibodies and traditional chemotherapeutic agents has shown a favorable clinical response and long‐term clinical benefits in patients with advanced esophageal cancer, as reported recently in KEYNOTE‐590^3^ and ESCORT‐1st study^4^ irrespective of the intrinsic PD‐L1 expression levels. We administered anti‐PD‐1 antibody tislelizumab in combination with albumin‐bound paclitaxel and nedaplatin as the first‐line treatment in an ESCC patient. The tumor regressed significantly after two cycles of treatment and even sustained remission during the subsequent 3 months without any anticancer treatment after occurrence of the fistula. The latter response was attributed to the tailing effect of ICIs, which has been reported previously.^7^, ^8^

The incidence of esophageal fistula is 4.7%–13.1% in patients with esophageal cancer,^6^, ^9^, ^10^, ^11^, ^12^ and 28.8%–33.3% of the incidences of esophageal fistula are related to tumor regression after treatment.^6^, ^9^ With the improvement in the objective response rate (ORR) of ICIs treatment for esophageal cancer, the rate of tumor regression‐related incidences of fistula may increase. Risk factors of esophageal fistula consist of progression to an advanced stage of cancer, involvement of the upper‐mid thoracic esophagus, occurrence of a large‐sized tumor, initial airway involvement, esophageal stenting, and prior administration of esophagus radiotherapy.^6^, ^9^ A retrospective study has previously reported the median time of development of a fistula to be 7.9 months from the time of initial diagnosis of esophageal cancer.^9^ However, we believe that the time depends on the response of the tumor to treatment. The patient reported in this case study developed TEF on the 41st day after initiation of treatment.

Treatment of ESCC‐related TEF is usually palliative, involving esophageal intubation, stent, or surgery, such as surgical resection/repair of the fistula, esophageal bypass, or gastrostomy/jejunostomy.^6^, ^10^, ^11^, ^12^ Due to a relatively long segment and obvious remission of the tumor, and cancer involvement of the upper‐mid thoracic esophagus, it was speculated that there were high chances of stent displacement and surgery was not a practical and feasible option as discussed and decided upon by the MDT. A nasointestinal tube was implanted in the patient to ensure enteral nutrition. TEF is often associated with poor survival and the median survival time after diagnosis of TEF is 2–5 months.^6^, ^9^, ^11^ A previous study has reported that the survival period of a patient with implanted intubation or stent is longer than that of supportive therapy,^11^ and an esophageal stent improves overall survival in patients with malignant esophageal fistula compared with feeding gastrostomy/jejunostomy.^10^ To date, the patient in this report has survived for more than 16 months after the occurrence of the fistula without any sign of cancer progression or esophageal rupture.

During SAEs the use of ICIs may be completely stopped. Once the patient totally recovered from the AEs and his health status significantly improved, an ICI‐based rechallenge may potentially control the tumor however, but this may also increase the risk for recurrent immune‐related AEs. It is vital and difficult to maintain the safety‐efficacy balance of an ICI‐based rechallenge. A systematic review showed that patients who had previously discontinued ICIs due to toxicity‐related side‐effects achieved better tumor regression than those who had stopped treatment due to disease progression.^13^ The incidence of AEs in patients rechallenged with ICIs was similar to that reported for patients initially treated with ICIs.^13^ Several studies showed that ICI‐based rechallenge improved survival in cancer patients,^14^, ^15^, ^16^ and the subsequent AEs were generally manageable,^14^, ^15^, ^16^ but their onset was sudden and quick when compared to the original AEs.^14^ To mitigate the incidence of recurrent toxicity, some researchers adopted a strategy of ICI‐based rechallenge in combination with prophylactic immunosuppression, but the efficacy remains controversial.^13^

For the patient reported in this study, the occurrence of TEF was attributed to the high efficiency of the treatment administered, and the ICI‐based rechallenge could potentially lead to the recurrence of fistula or occurrence of other fatal AEs, but may potentially lead to an increase in the survival. The decision to initiate an ICI rechallenge in the patient was rather complicated. The optimum treatment duration of ICIs for advanced or metastatic cancer patients is up to 2 years as reported in clinical trials conducted previously.^17^ Data about the adequate duration of ICI‐based therapy in cancer is lacking.^18^ In the context of metastatic NSCLC, the CheckMate 153 trial reported that continuous ICI‐based treatment versus one‐year fixed duration of treatment improved survival, regardless of the response to treatment (complete response or partial response versus disease progression).^19^ The trial suggested that treatment with ICIs for 1 year is insufficient in most cases, so it was necessary to rechallenge with ICIs in the patient reported here who only received two cycles of ICIs before the occurrence of fistula. The durable response of ICI‐based treatment in cancer was attributed to the formation of memory T cells in the tumor microenvironment (TME).^20^ The PD‐1 molecules remain on the T cells for about 3 months.^21^ The half‐life of tislelizumab is 26 days. Therefore, ICI‐based rechallenge was reasonable after 6 months of the initial ICI‐based therapy.

This case report has certain limitations. The PD‐L1 expression and tumor mutation burden were not estimated, which may represent a major study limitation. Moreover, the ICI rechallenge analysis was based on data from an individual case, which may lead to biased conclusions. Prospective studies are needed to clarify the role of ICI‐based rechallenges administered after recovery from AEs in ESCC patients to obtain a more comprehensive insight into this potential therapeutic modality.

In conclusion, our results suggest that ICI‐based rechallenge in ESCC patients who have recovered from fatal AEs may serve as a clinically viable treatment strategy.

## CONFLICT OF INTEREST

The authors report no conflict of interest.

## Supporting information


**Supplementary Material S1** Upper gastrointestinal radiography at the time of fistula happened
**Supplementary Material S2**. Upper gastrointestinal radiography after the improvement of fistula
