# Supplementary material for: Successful immune checkpoint inhibitor‐based rechallenge in a patient with advanced esophageal squamous cell cancer: A case report
Source: Thorac Cancer. 2022 Jan 11;13(3):497–501. doi: 10.1111/1759-7714.14279 (PMC8807265; doi:10.1111/1759-7714.14279)
Supplement: Supplementary file 1 — Supplementary Material S1 Upper gastrointestinal radiography at the time of fistula happened Supplementary Material S2. Upper gastrointestinal radiography after the improvement of fistula [file TCA-13-497-s001.docx]

Supplementary material legends

Supplementary material 1. Upper gastrointestinal radiography at the time of fistula happened

Supplementary material 2. Upper gastrointestinal radiography after the improvement of fistula
